# Tinkering and the Origins of Heritable Anatomical Variation in Vertebrates

**DOI:** 10.3390/biology7010020

**Published:** 2018-02-26

**Authors:** Jonathan B. L. Bard

**Affiliations:** Department of Anatomy, Physiology & Genetics, University of Oxford, Oxford OX313QX, UK; j.bard@ed.ac.uk

**Keywords:** anatomical change, evolutionary change, developmental process, embryogenesis, growth, mutation, patterning in embryos, protein network, systems biology, variation

## Abstract

Evolutionary change comes from natural and other forms of selection acting on existing anatomical and physiological variants. While much is known about selection, little is known about the details of how genetic mutation leads to the range of heritable anatomical variants that are present within any population. This paper takes a systems-based view to explore how genomic mutation in vertebrate genomes works its way upwards, though changes to proteins, protein networks, and cell phenotypes to produce variants in anatomical detail. The evidence used in this approach mainly derives from analysing anatomical change in adult vertebrates and the protein networks that drive tissue formation in embryos. The former indicate which processes drive variation—these are mainly patterning, timing, and growth—and the latter their molecular basis. The paper then examines the effects of mutation and genetic drift on these processes, the nature of the resulting heritable phenotypic variation within a population, and the experimental evidence on the speed with which new variants can appear under selection. The discussion considers whether this speed is adequate to explain the observed rate of evolutionary change or whether other non-canonical, adaptive mechanisms of heritable mutation are needed. The evidence to hand suggests that they are not, for vertebrate evolution at least.

## 1. Introduction

The current, standard model for speciation is based on the modern evolutionary synthesis. This states that a new species arises from a subpopulation of an existing species that becomes reproductively isolated and finds itself subject to novel selection pressures. Differences between the two populations will arise if, as a result of such selection, a subgroup of existing anatomical, physiological, or behavioural variants in that subpopulation now start to produce more fertile offspring than they did, and relatively more than those organisms more typical of the original population. Under these circumstances, such variants will in due course become the norm. If the original and new populations remain reproductively separated, they will continue to diverge not only as a result of genetic drift and mutation, but also of restricted gene flow and divergent selection. Eventually, interbreeding between the two populations will no longer produce fertile offspring and a new species will have formed from a subpopulation of the original species.

**Figure 1:**
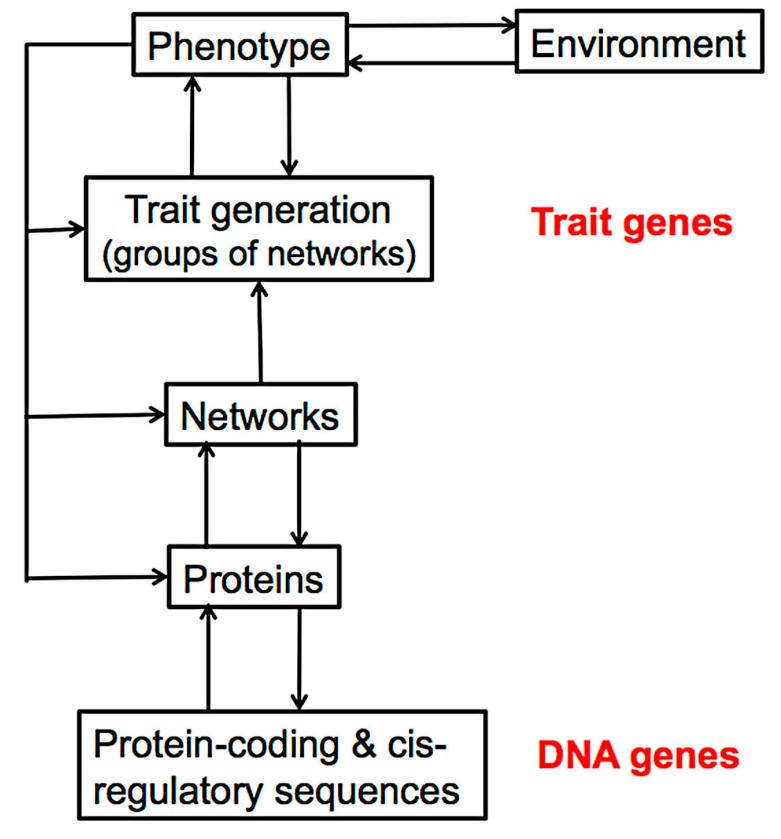
Diagram showing some of the levels of activity that link the genotype to the phenotype and to the environment, together with some possible feedbacks between them. The levels at which trait and DNA genes operate are shown in red. (From [9]).

The model thus involves mutation, variation, selection, and population genetics, with the least-well understood component still being variation. In 1859, of course, almost nothing was known about the basis of anatomical variation and Darwin could only write “Whatever the cause may be of each slight difference in the offspring from their parents—and a cause for each must exist…..” [1]. Even today, things are not clear for three main reasons, each of which makes experimentation difficult. First, as Huxley pointed out to Darwin [2], changes in adult anatomy mainly reflect events taking place during embryogenesis, and we still understand few of the molecular details of organogenesis in vertebrate embryos. Second, we know little about how mutation can affect the developmental phenotype in minor ways to give the normal spectrum of phenotypic variants. This is because the effects of that mutation have to work their way through a series of levels from proteins and their regulation, to the protein networks that drive change in embryos, and on to the cooperative and complex tissue interactions that underpin early organ formation and later anatomical change (Figure 1). Finally, it is very hard to identify minor anatomical or other variants in an embryo.

There is a further problem: evolutionary change is inevitably slow due to its random nature, the time taken for a potentially successful, novel mutation to spread across a population through genetic drift, and for the appropriate downstream adaptive changes to the phenotype to be selected [3]. Change would be much faster were there some direct feedback to the genotype from successful environment-induced phenotypic adaptation rather than it simply depending on natural selection of existing phenotypic variants. This general idea of heritable adaptive change was put forward by Lamarck at the beginning of the 19th century and accepted by Darwin and other 19th century biologists. However, it was disproved experimentally by Galton (1871) and by Weismann’s discovery in 1892 of the continuity of the germplasm and the absence of feedback from soma to germplasm, in animals at least (for historical summary, see [4]. Nevertheless, the idea that generating adaptive anatomical variants requires something more than just chance mutation refuses to die [5]: some workers still feel that the vast amounts of sequence and polymorphism data, phylogenetic analysis and coalescence work that explain so much of evolution [4] do not explain, in particular, the speed of change and recent evidence on environment-induced heritable change (see discussion). Thus, in addition to the problems with current views on selection, the main topic of this issue, there are also difficulties in understanding variation. This paper considers the origins of heritable anatomical change in the context of canonical mechanisms of variation, with the general intention of trying to understand whether vertebrate change needs to be as slow as generally thought and focuses on three themes. The first is that tissue formation derives from the activity of the many complex protein networks that drive anatomical change in embryos; these include the regulatory networks for signalling, patterning, and timing, together with the process networks that effect morphogenesis, proliferation, apoptosis, and changes to cell differentiation. The second is that anatomical variation derives from mutations that affect the expression, regulation, and properties of proteins that affect the output of these networks. The third is that the route from mutation to anatomical change involves events at several levels of scale. The approach is thus firmly within a systems-biology context [6].

What is not discussed here is plasticity, the existing ability of an organism to adapt its anatomical detail or its behaviour as a result of the environment in which it develops and lives—an obvious source of phenotypic, but not heritable, variation. This concept has been invoked as a mediator of evolutionary change and discussed in some detail by Gilbert & Epel [7], and it seems more likely that plasticity is a buffer against selection. If an organism’s genetic endowment results in it being able to adapt through, for example, camouflage or strength, and so become fitter in some environment, the selective forces to which it is subjected will inevitably be weakened and there will consequently be little pressure for heritable change: plasticity represents genotypic and evolutionary stability, rather than a force for change.

The paper starts by considering what can be deduced about the nature of variation from the anatomy of living and extinct vertebrates, particularly in the context of developmental processes. The analysis shows that the major evolutionary changes in anatomical detail mainly reflect changes in the activity of the pattern-formation, timing, and growth networks [8,9]. The paper then considers how these networks can be affected by mutation. The discussion covers the questions of the time needed for change to become established and whether processes other than mutation and genetic drift are needed. In terms of Jacob’s classic analysis, this paper explores what is involved in “tinkering” [10]. The general conclusion is that anatomical variation in vertebrates based on the existing genetic spectrum of variants is easier to achieve and can occur more rapidly than is generally supposed. Hence, there is, as yet, no need to invoke non-canonical evolutionary mechanisms for driving anatomical change here.

*A note on nomenclature.* The term *gene* is used in two very different contexts in evolutionary biology. The original one, which dates back to Mendel, defines a gene in terms of its direct effect on a phenotype. The modern one, which dates from around 1960, defines a gene as a DNA sequence with some function. The former is particularly used in evolutionary population genetics because it underpins behavioural, physiological, and anatomical traits, and can be assigned a selection coefficient. Such “trait” genes can however rarely be defined at the level of the genome as they reflect events at a far higher-level (Figure 1). In this paper, a gene is assumed to be a DNA sequence rather than having a role defined by the phenotype. 

## 2. Anatomical Variation in Vertebrates

The purpose of this section is to identify those anatomical processes in which mutation-induced change can lead to anatomical variation.

### 2.1. Variation within Crossbreeding Populations

**Figure 2:**
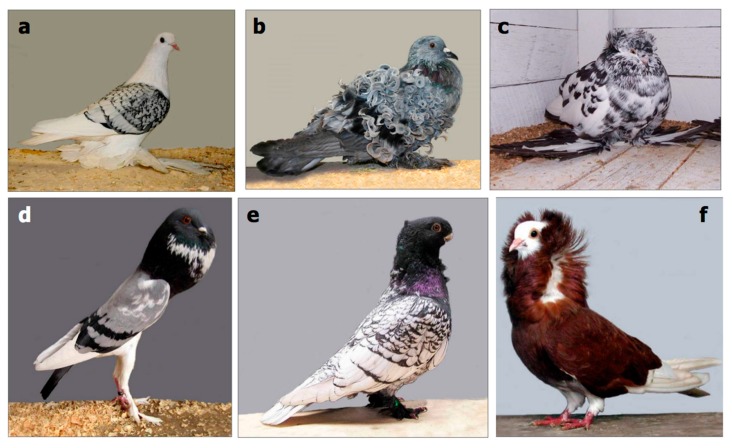
Pigeon strains. (**a**) ice pigeon; (**b**) frillback pigeon; (**c**) English trumpeter pigeon; (**d**) pigmy pouter pigeon; (**e**) oriental frill pigeon; (**f**) the capuchin red pigeon. From www.mnn.com/earth-matters/animals/stories/18-most-bizarre-pigeon-breeds (Courtesy of Jim Gifford, published under CC BY-SA 2.0).

Variation is particularly important in this context as it provides the basis of novel speciation. There is a spectrum of phenotypes in any group of organisms, partly due to minor genetic differences and partly as a result of adaptive plasticity. The classic example, because of the ease with which heritable novelties can be produced through selective breeding [11], is the range of feather patterns in pigeons, a topic that particularly interested Darwin (Figure 2). More wide-ranging in phenotype is variation within the Canidae, a group that includes domestic dogs, grey wolves, coyotes, dingoes, and golden jackals, each of which has 78 chromosomes and can interbreed with the others [12]. The extent of anatomical variation in this group is large: they can range in size from a Chihuahua, which is about 20 cm high and weighs about 2 kg, to a Great Dane which is about 75 cm high and weighs about 75 kg. Canidae can have plain, dappled, or spotted hair patterns, a wide range of colors, and ears with a considerable shape range. Their skeletons show a particularly wide degree of variation in the relative sizes of mandibles and skull length [13]. In addition, wolves have 42 teeth and dogs have 44.

The most striking anatomical differences among the Canidae reduce to variation in absolute size and relative proportions, hair pigmentation, and tooth number. In terms of the underlying molecular processes, these involve the regulation of growth, pigmentation, and numbering. It is significant that each is under the control of the patterning mechanisms that regulate the later stages of embryogenesis, well after the basic geometry of the embryo has been laid down. It is also interesting that both tooth and pigmentation patterning reflect mechanisms that regulate neural crest differentiation [14]. Little seems to be known about the molecular origins of these anatomical differences, other than that FGF8, a signal protein, may play a role in regulating the size of facial bones [15].

There is of course information from other groups and an interesting example that shows the importance of enhancer and other regulatory sequences in generating variants comes from analyzing pelvic-spine formation in sticklebacks. Marine sticklebacks have pronounced pelvic spines for protection; freshwater sticklebacks lack these spines and reflect a variant occasionally seen in marine sticklebacks [16]. Molecular analysis has shown that pelvic-spine formation requires local Pitx1 activity, a transcription factor whose wide expression is controlled by four enhancer regions. In freshwater sticklebacks, the enhancer region for the pelvic region has been lost and the gene is not expressed there [17,18]. As a result of this mutation in the patterning networks, the downstream module of signaling and process networks that are responsible for spine production is not activated.

### 2.2. Variation Across Non-Breeding but Related Vertebrate Groups

The range of species here is so wide that some focus is needed. A glance through any zoology book (e.g., [19]) makes it clear that the most obvious areas where there is extensive variation across related species include size, both absolute and relative, structures that vary in number and pigmentation patterns. As discussed below, such variation can also derive from changes that reflect developmental timing.

#### 2.2.1. Size

**Figure 3:**
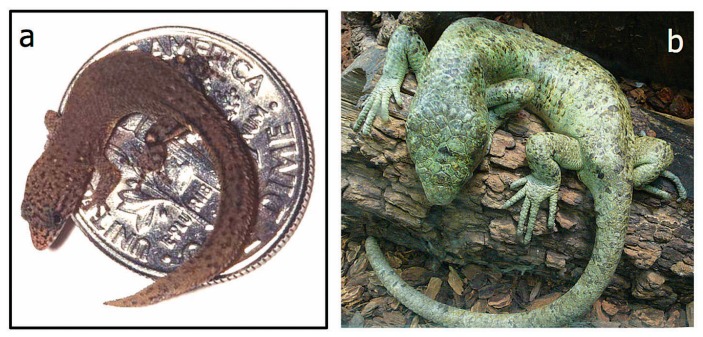
Small and large lizards. (**a**) *Jaragua Sphaero*, the dwarf gecko (from www.popsci.com/scitech/article/2002-01/small-beautiful, courtesy of S. Blair Hedges), is 16 mm long; (**b**) the Solomon Island skink (from en.wikipedia.org/wiki/Solomon_Islands_skink, courtesy of Dry Tim Vickers) is 830 mm long. The only obvious differences are in pigmentation and size.

A striking comparison here is between the dwarf gecko and the Solomon Islands skink: both of these lizards have very similar proportions, but the former is about 16 mm while the latter is about 800 mm long (Figure 3 [20,21]). This suggests a difference due to about five or six cell divisions, most of which reflects post-hatching growth. Here, the major difference is clearly is in the timing of when the body-wide growth networks cease activity. An example of the differences in relative sizes of a homologous tissue is the mandible: in humans, it is about 17.5 cm, or about 10% of the height, whereas the equivalent ratio for baleen whales is about 25% of the length [22]. 

It is worth noting here that proliferation rates are often under local control and can vary within a single developing or regenerating organ. This can be by a factor of about five across developing chick limb mesenchyme [23], and of about 70 in regenerating as compared to normal adult rat liver (a mitotic rate of about 3.5% rather than 0.05%; [24]). There are thus major opportunities for mutation to lead to a local change in growth rate.

#### 2.2.2. Number Variants

Numbering differences give a quantitative perspective, and obvious examples are in vertebrae, teeth, digits, and skin-pattern stripes. Vertebrae derive from somites, and the range in number within a single group is wide: in fish, for example, zebrafish have only twenty six vertebrae whereas american eels can have over a hundred. The record is, however, probably held by pythons that can have as many as 400 vertebrae [25]. The details of vertebrate morphology are controlled by Hox patterning and vary across the major families: there are, for example, seven neck vertebrae in mammals and 13–25 in birds (e.g., 14 in the chick), all being determined by the transcription factors of their specific Hox 3 and 4 paralagous groups [26,27]. The details of how this happens remain obscure.

Somite production is regulated by a clock-wavefront timing mechanism, with the amount of growth in the pre-somitic mesenchyme determining somite number. In most vertebrate embryos, the presomitic mesenchyme shrinks and is lost after producing something under 40 somites. Snakes have two adaptations here: their clock-wavefront mechanism produces somites at an unusually fast rate, while their presomitic mesenchyme is maintained for much longer than in other vertebrates [28].

Teeth numbers also vary, with the greatest range being seen in fish: sharks have a very large numbers of similar, replaceable teeth, while seahorses have none [29]. Variation is less in mammalian species as they typically have about 30–40 teeth, although pangolins and some whales have none. What is noteworthy is the range of tooth forms: these vary in size and complexity from incisors to molars to tusks. Most curious here are narwhales: they have one or occasionally two long tusks that erupt from their maxilla, sometimes together with minor, apparently randomly organized, vestigial teeth [30]. It is now clear that the mechanisms responsible for tooth production and morphogenesis are extremely complicated, involving at least five signaling pathways [31]. The details of how tooth-number variation is achieved across the vertebrates are still not known. 

A particularly visible example of number variation is in existing vertebrate autopods: digit numbers vary from one to six, with the standard number being five. The diminished range of one to four digits seen, for example, in horses, sloths, birds, and amphibians, reflect reductions in the eventual numbers of apoptotic zones seen in the developing autopod as compared to the four seen in the standard pentadactyl limb [32]. They thus reflect changes in the early patterning system that determines digit number, an event that precedes the later processes that control digit detail (e.g., phalange number, joint morphogenesis, claw differentiation and size). 

**Figure 4:**
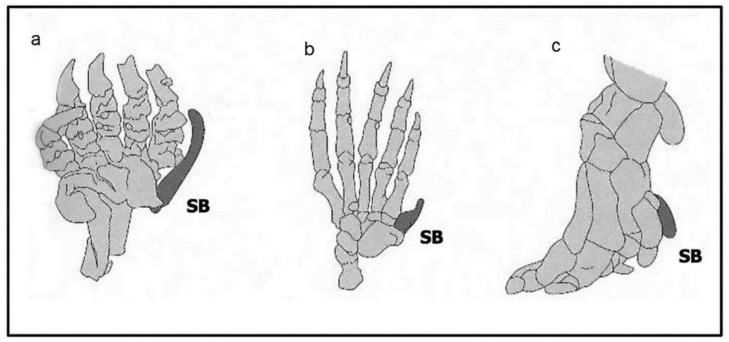
Ectopic digits. Drawings of the forelimb skeletons of (**a**) the mole; (**b**) the panda and (**c**) the elephant. Each ectopic digit (darkened) reflects unusual growth of a normal sesamoid bone (SB). (Courtesy of (**a**) Royal Society [33]; (**b**) Garland Press [3]; (**c**) Science [34]).

The sixth digit is different. This unusual feature is a homoplasy seen in three unrelated mammals: in mole forelimbs where it facilitates digging, in panda forelimbs, where it is used for stripping bamboo, and in elephants where it may increase limb stability (Figure 4, [33,34]). These three cases have two points in common: first, the additional digit reflects an enlarged radial sesamoid bone (it has no separate phalanges or nails), rather than a full repatterning of the autopod, and, second, this feature is also seen in each hindlimb although it has no obvious function in pandas and moles. These sixth digits clearly reflect a variant in late patterning that just changes the growth of both fore- and hind-limb radial sesamoids in these very different species. 

**Figure 5:**
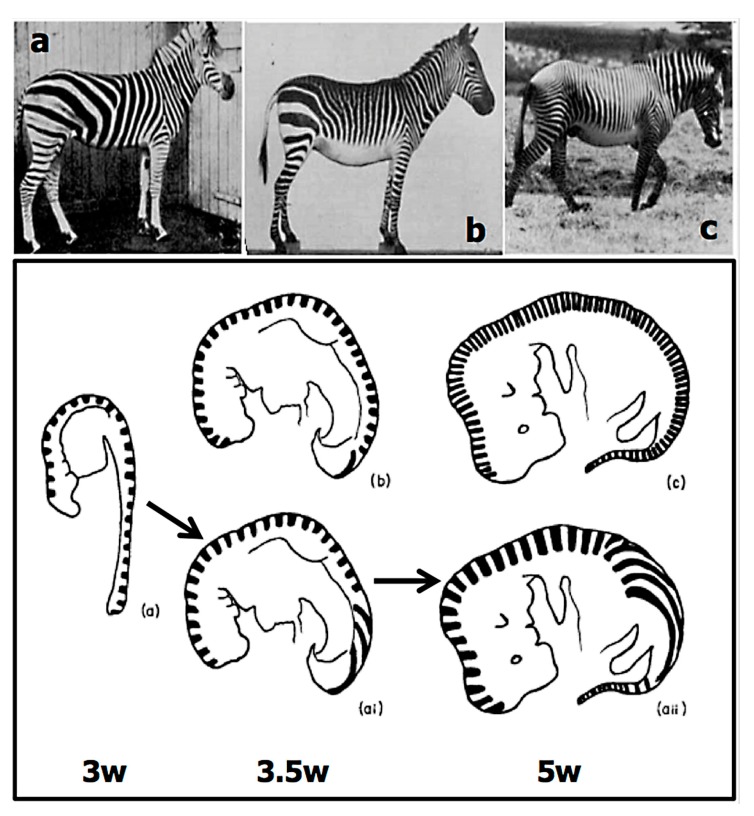
Likely initial patterning of zebra stripes. Top panel. (**a**) *Equus quagga burchelli*; (**b**) *Equus zebra*; (**c**) *E. grevyi*. Lower panel. Drawing of horse embryos aged 3, 3.5, and 5 weeks. The upper three drawings have stripes spaced at 200 µm, the lower two drawings indicate the effect of growth on the stripes that would have been laid down at 3 weeks. (From [35]).

As to number variation in pigmentation patterns, the classic example is the body striping number in the three zebra families: there are ~25 stripes on *Equus quagga burchelli*, ~40 stripes on *E. zebra* and ~75 stripes on *E. grevyi* (Figure 5, upper panel). Analysis of horse development during early embryogenesis suggests that patterning in the neural crest cells that will form the black and white pigmentation in the hairs of the stripes is laid down at about 3, 3.5, and 5 weeks of development in the different species [35]. It is also intriguing that horse-zebra hybrids have more, but thinner stripes than their zebra parent; this again probably reflects delayed patterning in their embryos. Pattern variants thus reflect timing variants. It is also noteworthy that there is a strong random element in zebra stripes: even the patterns on the two sides of a single animal are different.

The mechanism generating zebra stripes is not known, but there is strong evidence that they are the result of reaction-diffusion (Turing) kinetics, as discussed in the next section. It is interesting in an evolutionary context that such kinetics can also generate the complex patterns of color seen in fish scales [36], and the hair patterns of giraffes and cats [37]. The key point is that minor variation in a basic patterning system can produce a wide range of patterns.

### 2.3. Variation in the Fossil Record

The importance of the vertebrate fossil record in the context of variation is that it can show the stages in the progression of an early tissue to a later homologue (Darwin’s descent with modification), and an example for which there is good fossil data is the transition of the fish fin to the pentadactyl limb. Table 1 shows some of the key organisms that demonstrate the changes that led from a mid-Devonian (c. 395 Mya) sarcopterygian fish, such as *Kenichthys* to *Perdepes*, a primitive amphibian from the early Carboniferous period (c. 348 Mya) able to walk on land. The former had pectoral and pelvic fins that included a single-boned stylopod and a dual-boned zeugopod from which cartilaginous rays extended; the latter had digits rather than rays and proximal bones whose shape had been repatterned. The full transition from sea to land also involved, of course, the evolution of the pectoral girdle from fin-supporting bones, the full pelvic girdle from rudimentary pelvic bones, the expansion of lungs, and the loss of gills [38]. 

The fossil data thus show that the main evolutionary changes involved in forming the amphibian limb were in the patterning rather than the process networks. Amphibian limb development required nothing that was anatomically new: long bones, growth plates, and joints, together with their associated cell types were already present in other parts of the skeleton, while apoptosis is part of the fish developmental repertoire [48]. The key requirements were a set of repatterning changes that removed rays, produced digits, remodeled bones, and modified growth. Particularly interesting in this context was the morphology of *Sauripterus,* which had both digits and rays [41]. When the latter were lost, there was a range of digit numbers in different fish before the pentadactyl limb became the norm [40].

The information in Table 1 also indicates how long the transition took. Although it is rarely possible to be certain when a species was first established, the data to hand suggest that it took some 20 My for the weight-bearing limb with digits to evolve from a fin. The selection pressure that drove the transition was probably the ability for a fish to thrive in shallow waters with dense plant life [38]. Here, an ability to brush plants aside with ever more powerful fin appendages to obtain food could have represented a selective advantage. Nevertheless, the degree of anatomical change that must have occurred over each million (and probably as many generations) of the 20 My transition period was clearly relatively minor.

The transition from sea to land also of course involved the production of the pectoral and pelvic girdles and the change from a gill-based respiratory system to one based on lungs [38]. Here, it is worth noting that these changes required anatomical repatterning rather than the evolution of new cell types: oxygen-uptaking cells, for example, had to have been present in both the gills of fish and the primitive lungs of dipnoi.

### 2.4. The Experimental Data on Variation

It has long been known that there is a very great deal of variation in all normal populations, arising through the slow background mutation rate, with both old and novel mutations being distributed among the population by random breeding—this is genetic drift. It has however been difficult to study the potential effects of extant variation in vertebrates experimentally and the only practical model organism is *Drosophila*. This is partly because of our knowledge of its genetics, partly because of its short breeding time and partly because of its cheapness: these experiments need many thousands of animals. Here, there are two classic sets of experiments that set out to generate novel phenotypes from populations of wild-type flies: the work of Rice & Salt [49] on driving the early stages of speciation in *Drosophila* and of Waddington [50] on generating *Drosophila* with the *bithorax* phenotype. 

Rice and Salt subjected a wild-type population of newly hatched flies to a series of preferences: light/dark, ethanol/acetaldehyde, and up/down, selectively breeding flies with the same preferences. After some 35 generations of inbreeding, they had two distinct fly populations with a set of opposite preferences that would not breed with one another. In generating these behavioral variants, they achieved the first stage of sympatric speciation, although the genetic basis of the variants is still not known. 

**Figure 6:**
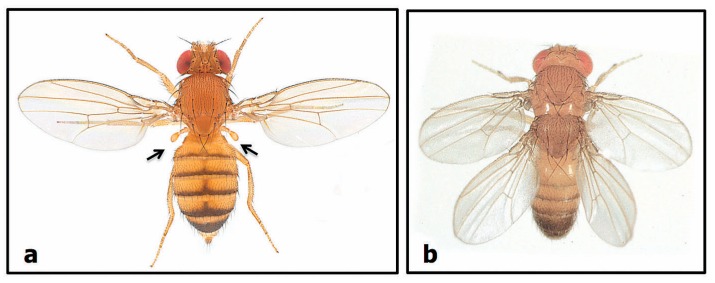
The *Drosophila* bithorax mutant. (**a**) A normal fly with two wings and two halteres (arrows); (**b**) The bithorax mutant which has a duplication of the second thoracic segment instead of a normal third one: it therefore has a second pair of wings and no halteres ((**a**) Courtesy of Nicholas Gompel ©; (**b**) www.researchgate.net/figure/Four-winged-fly-produced-by-combining-bithorax-and-postbithorax-mutations-Reproduced_fig1_240636076 Courtesy of Steve Carr).

Waddington’s experiments started from the observation that fly embryos exposed to ether occasionally displayed the radiation-induced bithorax phenotype, which is characterized by a duplication of the second thoracic segment at the expense of the third (Figure 6); this results in flies with four wings rather than two wings and two halteres (balancing organs). After 25 generations of ether treatment and selective breeding, Waddington obtained lines of four-winged *Drosophila* flies that bred true without ether treatment—the rare bithorax phenotype had been assimilated (to use Waddington’s word) into a normal, breeding population. It is however worth noting that these experiments only worked if done on wild-type fly populations with considerable amounts of natural variation. 

The implications of Waddington’s work, done before the molecular revolution, were originally unclear. Today, however, the experiments show that there were appropriate mutations distributed with low frequency across the original population that, if brought together by selective breeding, could produce a bithorax phenocopy, a form now known to be due to mutations in the bithorax complex and its regulatory sites [51]. A similar conclusion can be drawn from the work of Rice and Salt [48]. Although these two sets of experiments required extreme selection to achieve their rapid results, they emphasize that the extent of genetic variation in wild-type populations is sufficient to generate novel phenotypes showing behavioral and anatomical change, with change being relatively rapid if the selection pressures are high enough.

### 2.5. Implications of the Evidence on Variation

This brief summary of the data on variation leads to several conclusions. One initially surprising observation is the similarity in the types of differentiated cells across the various vertebrate families today; it seems that the main cell types all evolved early, and, given what we know of their anatomy, were present in mid-Devonian fish. Later additions probably included additional blood-cell types, osteoblasts, the various keratin-secreting cell types (e.g., for hair, horn and feather; [52]), and perhaps some novel pigmentation variants. Similarly, we can be fairly confident that there was little change in the apoptosis mechanism. Perhaps this stability of cell types is less surprising than it seems: a change of differentiation reflects a switching of states, and, if this were to go wrong, the result would probably be fatal, as is the case for the Runx −/− mutants that block osteoblast development [53].

Morphogenesis, which reflects the physical activity of cells (e.g., movement, adhesion, the folding of cell sheets) is, in principle, a quantitative entity and one can envisage changes in the speed of movement and the extent of folding. It is, however, hard to identify a modification in phenotype either across related species or from the fossil record that reflects variation in a morphogenetic process, as opposed to a change in the patterning of that process (e.g., the amount of time for which that process operates or the domain over which it operates).

The most dramatic examples of evolutionary change are thus, as the examples above have made clear, primarily in spatial patterning and in timing (heterochrony)—the where and the when of embryogenesis. Patterning changes lead to alterations in the processes operating within a specific domain, and hence to changes in local tissue geometry, differentiated state, growth rate, and shape (consider the differences in ear morphology across the vertebrates). Patterning is, however, an umbrella term that that also includes the results of simple and complex signaling, and indirectly lineage. Although this reflects immediate cell inheritance, it usually derives from signaling at an earlier stage of embryogenesis. Superimposed on all of this, of course, are the timing mechanisms that regulate when processes are activated or stopped.

The other process that is important is proliferation: mitotic rates can be modified, as the examples of liver regeneration and autopod development mentioned above show. Such growth can have secondary effects: the convolutions seen in the human brain cortex as opposed to, for example, mice result from neuronal proliferation: proliferation is far more extensive in the early brains of humans as opposed to mice, and the relatively increased size of the early cortical epithelium means that it has to buckle in order to fit inside the human cranium [54].

## 3. The Molecular Processes of Tissue Development

**Figure 7:**
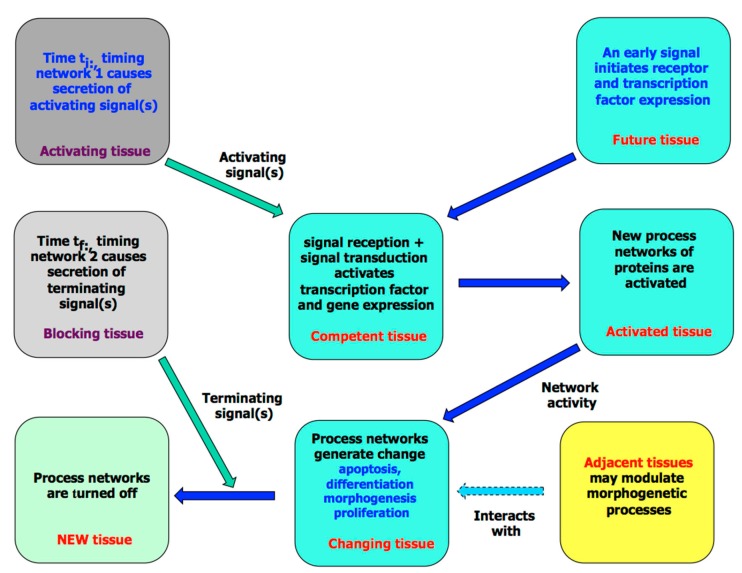
A diagram showing the various stages involved in the development of a simple tissue. Note that the events shown here assume that early signaling ensures that the activating tissue secretes one or more signals and that the future tissue is competent to receive it through expressing the appropriate receptors and transcription factors (this in turn depends on its lineage). Termination may be due to either external or autocrine signaling.

As anatomical changes in adults usually reflect changes made during development, any molecular analysis of variation has to start with organogenesis. This has two distinct stages. The first is tissue initiation: a rudiment first becomes competent through the expression of appropriate receptors and transcription factors, and it is then induced to develop by signals, usually from neighbouring tissues. Downstream signal transduction activates the process networks discussed above, and these in turn cause the tissue to develop further (Figure 7; for details, see [9]). This is the simplest mode of a patterning signal. Complex patterning leads to a distributed response, which may result in several tissues forming or in a single tissue having a graded phenotype. Well-understood examples here are the formation of the vertebrate neural tube and the *C. elegans* vulva [55,56].

**Figure 8:**
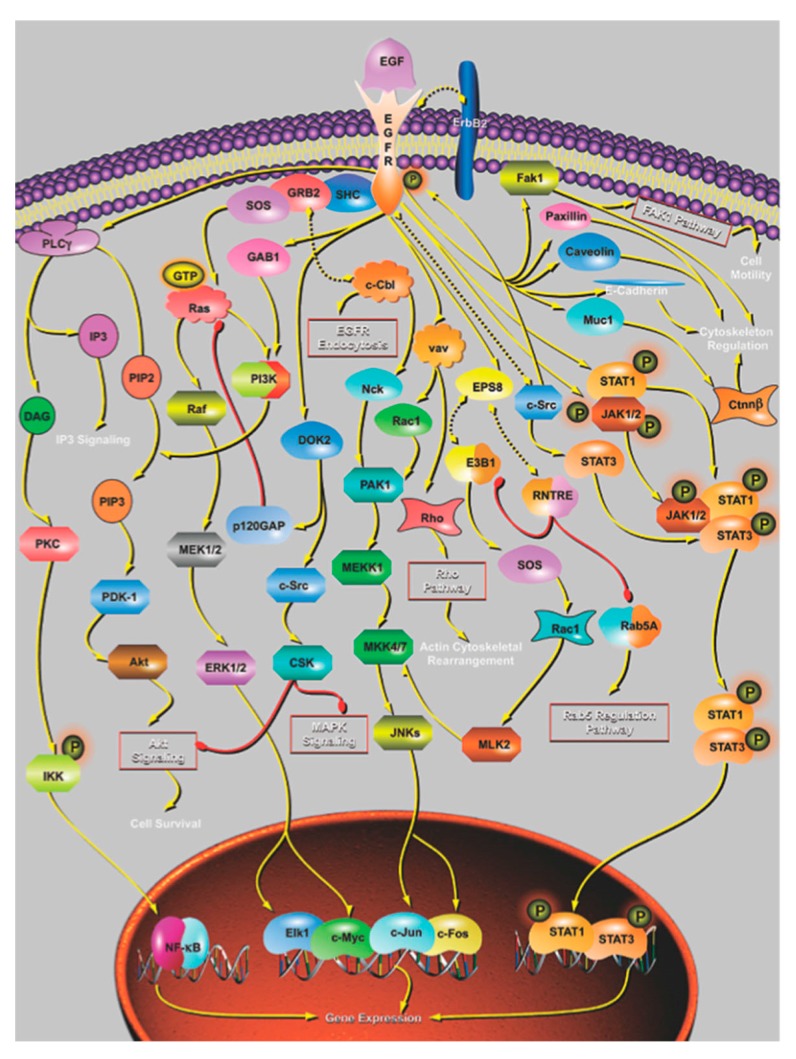
The epidermal growth factor (EGF) process network that activates, among other possible events, entry to the proliferation cycle contains ~50 proteins; this complexity is typical of many developmental networks. Very little is known of its internal dynamics or of the new proteins expressed following transcription-factor activation. (From Pathways Central, www.qiagen.com/us/shop/genes-and-pathways/pathway-details/?pwid=145, © 2009 QIAGEN, all rights reserved.).

Much is known about the molecular basis of proliferation and simple signaling, less about complex patterning and very little about the regulation of timing in embryos. Proliferation is normally initiated when one tissue secretes a signal protein that binds to a second tissue expressing the appropriate receptor for a growth network. A typical example is that for the epidermal growth factor receptor whose downstream network contains ~50 proteins (Figure 8). Such complexity is typical of many developmental networks (see www.sabiosciences.com/pathwaycentral.php), but the mechanism by which they all work is the same: cytoplasmic activity leads to the activation of extant nuclear transcription factors and a round of gene expression, which drives change. In this case, the effects include the initiation of the mitotic cycle. 

Even when their protein constituents are known, however, the internal dynamics of these networks are very hard to study. There has been some work on the relative importance of proteins with high and low rate constants (e.g., [57]), while Alon and his co-workers have shown the existence and importance of protein motifs, small groups of proteins that together have a single function and that might provide a means of simplifying the functional analysis of large networks [58]. Nevertheless, there is no case, other than for relatively simple signal-transduction pathways (e.g., [59]), where we understand the internal dynamics of the networks that drive development. 

The very few timing networks whose details are known are also complicated: that responsible for the formation of new somites at a rate of about one every 1–2 h somites involves the Wnt, FGF and retinoic acid signaling pathways [60,61]. This segmentation mechanism is however the prelude to the subsequent mechanism that sets up Hox patterning and so gives identity to each somite. The details here are still opaque, but a retinoic acid gradient plays an important role here. This is perhaps the only embryonic timing network some of whose details are understood; much more is known about the regulatory networks that drive the mitotic and circadian cycles [62,63].

There is also the reaction-diffusion (Turing) mechanism mentioned above. This mechanism is tissue-autonomous and assumes that each cell has the same pattern-forming protein network [64]. The system has two key features: first, it has at least two signaling molecules (morphogens), themselves active members of a complex network, that can diffuse through the tissue, and, second, there is an instability in the kinetics of the network, which, if reached, leads to an unexpected dynamic. Turing showed that, under these conditions, the signal molecules would form an energy-driven chemical-concentration pattern of peaks and troughs [64]. The exact form of the pattern depends on the details of the kinetics, the reaction constants, and the boundary conditions, but a key element is that the instability imposes a degree of randomness on the pattern [37].

Assuming that the morphogen pattern underpins the resulting tissue pattern, the kinetics can generate all forms of epidermal hair (e.g., zebras and cats) and fish-scale patterns in two dimensions [36,37], as well as the configurations of limb bones in three dimensions [65]. Although the patterning arguments in favor of such reaction-diffusion kinetics occurring in embryos are strong, supporting molecular evidence is still weak, mainly because it is very difficult to study Turing kinetics in vivo.

## 4. The Effect of Mutation on Proteins and Protein Networks

As is well-known, heritable mutation can occur in many ways from a single base change to duplications, through insertions (e.g., transposable elements) up to major genomic reorganisations of zygotic DNA [66]. Indeed, such complex mutations underpin the evolutionary origins of many anatomical features [67]. Nevertheless, if that mutation has no effect on protein expression or activity, it is essentially silent; indeed, in the context of evolutionary change, it is the effect on the phenotype that is of prime importance with the exact molecular reasons for this effect being secondary.

The effect of a mutation that alters the protein-coding region of a gene is relatively clear: the functional properties of the protein (e.g., its rate and binding constants) might or might not change, while the expression domain or splice-form might be altered. Indirect mutations, such as those that affect cis-regulatory elements, such as enhancers, although not always easy to locate, are particularly important here as they can affect the amount or even the expression of a protein-coding gene [68,69]. Both can be investigated by gene targeting, and it is as a result of such work that so much is known about simple signaling and its effects. There is a further point about the effects of mutation on anatomical development: if the mutation has a phenotype it will usually be indirect. The mutation will affect some aspect of protein function and this will have a secondary effect on a regulatory or process network that will eventually lead to an alteration in anatomical detail (Figure 1). Exceptions mainly occur when the mutation affects a protein such as collagen, which has a direct structural role.

Gene-targeting work has made it clear that a mutation that changes the switch effect of a single signal-receptor pair is rarely if ever beneficial; this is because there is no obvious redundancy in the system. Similarly, mutations affecting the outputs of the differentiation and apoptosis pathways are likely to be fatal, because such a mutation would either make the change in state less effective or lessen the regulation of that change (e.g., the Runx knock-out mutations that particularly block osteoblast differentiation [53]). It is also hard to see how a mutation that affects a morphogenesis pathway could be advantageous. 

The effects of mutation on the dynamics of a protein network with variable outputs such as that for proliferation (Figure 3) or patterning are much harder to work out; this is because we know so little about the internal dynamics and output properties of such networks. Altering the rate constant of an early-acting protein might activate an inappropriate route through the network, while changing that of a late-acting protein might affect the output of the network. 

In an evolutionary context, there is now a great deal of evidence showing that there is a considerable homology in process networks (see the KEGG pathway database http://www.genome.jp/kegg/pathway.html). This argues for there having been a great degree of internal stability with internal buffering against minor mutational changes in their individual proteins. Nevertheless, if a mutation has an effect that is too pronounced to be fully buffered, then the output of the network may well change, and this would generate a phenotypic variation that could be advantageous. Moreover, because of the richness of multi-protein networks, and the limited range of outputs of networks, we can envisage that mutations in several of the network’s proteins will lead to much the same result. It was for that reason that Wilkins [70] referred to networks as amplifiers of mutation.

There is a further implication in the fact that mutations in several genes can lead to the same change in the output of the network: the resulting alterations to the phenotype can be achieved more rapidly than those caused by mutations in a single gene. If the resulting phenotype has a selective advantage, there will, of course, be no difficulty in the underlying mutations being passed on to the next generation as they only cause variations in extant sequences.

## 5. Discussion

Anatomical development is activated by the outputs of the networks specifying patterning and timing, with these in turn activating the various process networks (for differentiation, apoptosis, morphogenesis, and proliferation) that drive local tissue construction. It is as if the genome included a set of subroutines that could be used in any context, with perhaps a degree of secondary tissue-dependent tuning. The analysis of vertebrate anatomical variants discussed in this paper shows that evolutionary change mainly derives from mutations that modify the networks specifying patterning, timing, and growth with minor mutations in them being responsible for normal variation. This is probably because mutations that modify the differentiation, apoptotic, and morphogenetic networks are unlikely to be beneficial and so be lost.

Beyond these straightforward conclusions are a series of problems. First, it is rarely if ever yet possible to identify the modes of action of the particular mutations that have been responsible for driving anatomical change. This is because it is still impossible to work out how a mutant protein alters the reaction kinetics of the network in which it participates and so changes its functional output. It is even harder to identify mutations that have neutral or potentially beneficial effects; experimentation normally identifies deleterious ones, which are recognisable by their abnormal anatomical phenotype [71]. 

Second, we still know very little about the details of the patterning and timing networks that drive normal development, let alone how mutation leads to changes in their outputs. In the case of timing, this can involve the altering the activation time for starting or stopping a developmental event (heterochrony). Keyte & Smith [72], for example, point to the relative lateness for stopping a process, such as somitogenesis in snakes as being a major driver of their lengthening, while timing changes are associated with the early development of marsupial forelimbs [73]. 

A deeper problem with timing is in coordination. For a patterning event to take place at a given time in embryogenesis, earlier decisions will have had to be made by both the responding and the pattern-inducing tissue. The former needs receptors and transcription factors to be in place, while the latter has to be ready to secrete signals (Figure 7). This of course means either that the tissue has been subjected to an earlier round of patterning or that the tissue includes autonomous lineage mechanisms that allow it to prepare for change. The only case where we have some insight into the molecular basis of a timing network here is the periodic mechanism that drives somitogenesis [61], and this seems unlikely to be used elsewhere in the embryo. If we do not understand the molecular basis of these mechanisms, we certainly will not know how they can be affected by mutation, although any mutants with an abnormal timing phenotype might be helpful probes into understanding normal development.

Third, and even more complicated are the problems that are associated with understanding how the effects of a mutation work their way upwards through the different levels of scale to tissues and eventually modify the complete organism. This difficulty is compounded by the fact that there is usually feedback between the different levels (Figure 1). Such events can have consequences that are often hard to understood, let alone predict (e.g., the effect of external temperature on the future sex of some reptiles [74]). Events at many levels lie between an initial mutation and a change in an organism’s anatomical phenotype that is selectively advantageous enough in a new environment to become the predominant form. This complexity makes it clear that there is unlikely ever to be a full theory of evolutionary change, one that is capable of making quantitative predictions.

### The Speed of Evolutionary Change

The fossil record gives some insight into the speed with which a novel advantageous anatomical variant spreads through a population as a result of genetic drift and natural selection. This is rarely fast: the evolution of most phyla, eukaryotic cell types, and forms of morphogenesis took place over the sixty million years between the late Ediacaran period and the end of the Cambrian Period (~560–485 Mya); change since then has been relatively slow. Even the relatively straightforward transition of the sauropterygian fish fin to an early amphibian limb seems to have taken almost twenty million years and probably almost as many generations (Table 1). Two obvious questions about the speed of any anatomical change are first whether it happened because the requisite mutations were already present in the population or whether novel mutations had to arise and second how high were the selection pressures—and we can rarely answer either.

Perhaps unexpectedly, the species about which we know most, mainly because so much work has gone into investigating its evolution, is our own. Consider the changes that have taken place since a last-common great ape group split some 6.5 Mya to give the separate lines that led to the panins and the hominins, with the latter eventually (c. 200 Kya), resulting in *Homo sapiens.* The main anatomical changes in the latter have, over the last 2 My or so, been the loss of much body hair, minor changes in growth, changes in skeletal morphology, notably the skull, and increase in the size of the brain, particularly the volume of the cerebral cortex [75]. While this latter increase clearly led to major improvement in mental capacity it is hard to point to any new anatomical tissues, other perhaps than Broca’s area that handles speech, which have formed over this period of more than 100,000 generations. Elsewhere, there are minor molecular differences that probably reflect novel mutations, such as in the variants of the epidermal differentiation complex where positive selection has been shown to exist in primates (e.g., *SPRR4*) and in humans (e.g., filagrin); even so, the time scale for change is still of the order of thousands of generations [76]. Anatomical change in the line that led to humans has not been particularly rapid, apart perhaps from the increase in size of the cerebral cortex. 

Further information on human evolution comes from the genetic and population data on the final major *H. sapiens* migration out of Africa ~65 Ky or 3–3.5 thousand generations ago ([77,78]; note that an *H. sapiens* emigration from Africa some 10 Ky earlier led to the indigenous population of Australia). Evidence from genetic and coalescence studies suggests that a founder group of 500–1500 breeding individuals left Africa across the Sinai peninsula, and in due course populated the world [79]. This group included most of the variation now seen outside Africa, apart from small contributions from the then extant Neanderthal and Denisovan populations. These were descendants of *Homo erectus* populations who had left Africa some 500–400 My earlier, and it is an interesting comment on the slowness of generating major chromosomal changes that they and *H. sapiens* were still able to interbreed. Today’s variants in *Homo sapiens* mainly affect pigmentation and minor changes in the facial morphology—there are no new anatomical tissues—and are relatively trivial in the great scale of evolutionary change [80].

The evidence from sequencing studies clearly shows that, the further a population is from Africa, the less is its genetic diversity [81]. Coalescent analysis based on DNA sequences points to a succession of small founder groups with unrepresentative gene variants migrating into new regions [79]. It is noteworthy, for example, that the group that crossed the Baring Straits some 15 Kya (~750 generations) and whose successors colonized America was unlikely to have included much more than a thousand breeding individuals. The implications here is that, if a population is small and includes an existing trait variant with a reasonable selective advantage (e.g., the relative lack of pigmentation in northern Europeans), then it may well take only a few hundred generations of genetic drift and natural selection for that trait variant to become part of the normal phenotype of the descendants of that initial population. Such evolutionary change can be much faster under experimental conditions of very strong selection: even if several mutations are required and they are present at low frequency in the population, a trait can become pronounced in only around thirty generations [49,50]. If indications of that novel trait already exist in a founder population, selection can make it become a normal feature of the population’s phenotypes in as few as ten generations [82].

This paper has pointed to a further aspect of breeding that reflects on the rate of change: mutations in several genes can change the output of multi-protein networks in the same way. Not only can this effect amplify the effect of mutation [69], but it can also speed up the effects of genetic drift, allowing mutant genes each of which may have a similar effect on the phenotype to independently move through a population. In short, change through normal mutation can happen quite rapidly on an evolutionary time scale, even though it is usually too slow to be detected, even in an organism like *Drosophila* with a reproductive cycle of only about two weeks.

Nevertheless, some still feel that speedier mechanisms of evolutionary change are needed [5] and various epigenetic solutions have been suggested by which an environment-induced phenotypic change in a physiological system would feed back to the genome of the relatively isolated germ cells, particularly the oocytes that are laid down during development [6]. The first was pangenesis, Darwin’s idea [83], that sperm and eggs included contributions from functioning tissues, an idea that was disproved by Galton (see [84]). A more recent suggestion is that, because DNA methylation can be functionally induced, this may provide a mode of epigenetic inheritance. This suggestion is unlikely because, in mice at least, genomic methylation in early germ cells, other than in the very few known cases of imprinting, is lost [85,86]. More recently, Iqbal et al. [87] have shown that other parental methylation in pregnant mice is lost within two generations.

There is however some evidence of transgenerational epigenetic change that cannot be explained by normal mechanisms of mutation, such as through transposon mediation [88,89,90], although its significance in generating anatomical change has yet to be demonstrated. The difficulty is of course in directing adaptive change in somatic cells back to the germ lines, something that is hard to envisage in animals. The problem is however lessened in organisms such as fungi and plants where the generation of somatic and germ lines is much less distinct, with the latter often forming from mature tissue [91].

The best-known examples of successfully induced heritable change that have lasted at least forty generations have occurred in *C. elegans* that have been fed bacteria expressing a dsRNA that targets one or another regulatory gene. In these cases, it is clear that the RNA has been assimilated into the genomes of the reproductive cells of this very small organism, but it is unlikely that this is a common or even a realistic mechanism for normal evolutionary change (for review, see [7]). 

This paper thus confirms the consensus view that, in seeking to explain the evolution of novel anatomical changes that emerge in vertebrates over evolutionary time scales, there is as of yet no compelling reason to invoke mechanisms other than those of the modern synthesis (e.g., [92]). This is that novel phenotypes that are subject to natural and other forms of selection can be produced by random, undirected mutations of many sorts spreading through a population by genetic drift. The paper has however tried to show that the ways in which these mutations can lead to novel phenotypes are more complicated but faster than has generally been appreciated. 

## Figures and Tables

**Table 1:** Steps in the transition from sea to land—some key organisms and the possible process changes they represent.

Name	Mya	Family	Novel Features	Changed Processes?	Ref.
*Kenichthys*	c.395	sarcopterygian	Earliest fish with nostrils linking to the oral cavity. Normal sarcopterygian fins with short limb bones.	Open nostrils due patterning changes inducing morphogenesis and apoptosis?	[39]
*Eusthenopteron*	c.385	sarcopterygian	Fish with internal nasal adaptations and labyrinthodont teeth; the bones of pectoral and pelvic fins had growth plates for lengthening.	Repatterning of growth of teeth and of upper limb bones	[40]
*Sauripterus*	c.380	sarcopterygian	A fish whose fins had both radials and primitive digits	Repatterning of distal bone organization	[41]
*Panderychthys*	c.380	sarcopterygian	A fish with four unjointed digit-like bones and a tetrapod cranium	Repatterning of limb bones and cranium	[42]
*Tiktaalik*	c.375	sarcopterygian	A fish with amphibian features. The pectoral fin had basic wrist bone but rays not digits. It also had a flexible neck, lungs, and basic pectoral and pelvic girdles	Novel bones and repatterning of existing bones. Major patterning & morphogenetic changes that turned gills into lungs	[43]
*Ichthyostega*	c.374	labyrinthodont	An intermediate species with amphibian-type lungs, strong ribs and fore and hind limbs (7 jointed toes) + fish gills & tail − able to clamber on land	Pattern formation + numbering	[44]
*Acanthostega*	c.365	labyrinthodont	A very early amphibian with 8 forelimb & 7 hind limb jointed digits, non-weight-bearing forelimbs, and a complete pelvic girdle	Pattern formation leading to numbering changes + apoptosis of tail	[45]
*Tulerpeton*	c.365	labyrinthodont	This species had 6 jointed digits, powerful “wading” limbs, pectoral girdle, lungs and no gills. This was clearly an amphibian.	Pattern formation + numbering	[46]
*Perdepes*	c.348	1st land tetrapod	An amphibian with 5 (+1?) digits. Land-adapted feet	Pattern formation	[47]
